# Simultaneous knockdown of uPA and MMP9 can reduce breast cancer progression by increasing cell-cell adhesion and modulating EMT genes

**DOI:** 10.1038/srep21903

**Published:** 2016-02-24

**Authors:** Anuradha Moirangthem, Banashree Bondhopadhyay, Mala Mukherjee, Arghya Bandyopadhyay, Narendranath Mukherjee, Karabi Konar, Shubham Bhattacharya, Anupam Basu

**Affiliations:** 1 Molecular Biology and Human Genetics Laboratory, Department of Zoology, The University of Burdwan, Golapbag, Burdwan 713104, West Bengal, India; 2 Department of Pathology, Burdwan Medical College and Hospital, BurdwanWest Bengal 713104, India; 3 Department of Surgery, Burdwan Medical College and Hospital, BurdwanWest Bengal 713104, India.

## Abstract

In cancer progression, proteolytic enzymes like serine proteases and metalloproteinases degrade the basement membrane enabling the tumor cells to invade the adjacent tissues. Thus, invasion and metastasis are augmented by these enzymes. Simultaneous silencing of uPA and MMP9 in breast cancer cells decreased the wound healing, migratory, invasive and adhesive capacity of the cells. After simultaneous down regulation, cells were seen to be arrested in the cell cycle. There was a remarkable increase in the expression of cell to cell adhesion molecule E–cadherin, and decrease in Vimentin and Snail expression. In addition, there was a significant decrease in the expression of the stem cell marker Oct-4. In the breast tumor samples it has been observed that, tumors, expressing higher level of uPA and MMP9, express less amount of E–cadherin. It has also been observed that few tumors also show, Vimentin positive in the ductal epithelial area. Thus, our model can help for checking the aggressive tumor invasion by blocking of uPA and MMP9. Our present observations also give the concept of the presence of aggressive epithelial cells with mesenchymal nature in the tumor micro-environment, altering the expression of EMT genes.

Breast cancer is the most frequently diagnosed cancer and the leading cause of cancer related deaths in women worldwide. Approximately one-third of all the women with breast cancer develop metastasis^1^ and due to its metastasizing ability therapeutic strategies for the metastatic breast cancer are few. Metastasis of the cancer starts at the primary site of the tumor by invading and degrading the basement membrane and extracellular matrix (ECM)^2^. This invasive nature of the tumor cells is necessary for the metastasis.

In the process of cancer progression, tumor cells which starts to dissociate from the primary tumor invade into the neighboring tissue and transmit through the blood vessels and finally form colonies at a secondary site^3^ is related to cellular behavior ‘Epithelial-to-Mesenchymal Transition’ (EMT). During EMT, there is loss of epithelial markers like E-cadherin, α and β-catenin, cytokeratins and tight junction proteins like claudins and occludins. The loss of E-cadherin is regarded as one of the well-known hallmarks of cancer. On the other hand, the mesenchymal markers like the Snail, Slug, N-cadherins, vimentin, fibronectin, matrix metalloproteinase, integrins α_v_ and β_1_ and smooth muscle actin are increased^4^. EMT has also been reported to take part in promoting the stemness of the cancer cells. It has also been reported that in the normal breast cancer tissues and breast cancer cells, EMT induces stemness^5^. The transcription factor, Oct-4 is essential for maintaining the self-renewal in the embryonic stem cells and high level of Oct-4 expression is correlated with lymph node metastasis^6^.

The dislodging of the cells from the primary niche marks the aggressiveness of the tumor^7^. During invasion and metastasis, destruction of the basement membrane is a crucial step which requires the activation of the proteolytic enzymes^8^. The first step in the breakdown of the basement membrane is mediated by the proteases^8^. In several types of cancer, proteolytic enzymes such as the serine proteases and metalloproteinases play crucial role in the tumor invasion and their enhanced production contributes to tumor progression^8^. During tumor progression, urokinase plasminogen activator (uPA) after binding to its receptor uPAR, activates a cascade of proteases. The activated cascade of proteases leads to the degradation of the basement membrane^8^.

Many studies have been conducted on the relationship between uPA as well as MMP9 expression in cancer patients. In a variety of malignancies including breast, ovarian, glioma, lung, colorectal, gastric, thyroid and prostate cancer, uPA is over-expressed^2^,^8^,^9^. It has been observed that uPA was expressed at a high level in cholangiocarcinoma patients^2^. In the ovarian and breast cancer, uPA and PAI-1 have also been found to be expressed at a high level^10^. Elevated level of uPA was observed in various metastatic tumors and correlates with tumor aggressiveness^11^. Higher uPA level indicates reduced patient survival and act as prognostic marker along with PAI-1^11^,^12^.

The serine protease uPA when bound to its cell surface receptor uPAR not only converts plasminogen into plasmin but also activates the metalloproteases. Along with the plasmin, MMPs degrades the extracellular matrix^13^. The matrix metalloproteinases (MMPs) due to their proteolytic nature degrade proteins that regulate various cellular behaviors related to cancer cell differentiation, migration, invasion, and surveillance of the immune system^14^. In the breast cancer patients, high MMP9 expression is related to tumor stage and lymph node metastasis^15^. In addition, it has also been reported in the breast cancer patients that there is a significant association between high MMP9 expression and poor survival^15^.

The uPA/uPAR system induces the epithelial to mesenchymal transition signaling^16^. The MDA-MB-468 cells acquire mesenchymal character when uPAR has been over-expressed by hypoxia. The mesenchymal character is reverted back to the epithelial character after silencing of the uPA gene^16^. The role of the uPA/uPAR system in EMT was further supported by the demonstration on the intermittent hypoxic conditioned medulloblastoma DAOY cells. After silencing of the uPA and uPAR genes by siRNA in the DAOY cells, there was a significant decrease in the mesenchymal marker like the Vimentin with a subsequent increase of the epithelial marker like the E-cadherin^17^. It has been reported that E-cadherin, the important molecule of epithelial character, is a direct target of the MMPs including MMP-3, -7, -9 and MTI-MMP responsible for blocking the invasion^18^. In addition, EMT is also induced by the matrix metalloproteinases (MMPs) in cultured cells^18^.

In animal models, a reduction in metastasis has been reported after the inhibition of uPA alone or the inhibition of the interaction of uPA/uPAR complex^9^. In another study, antisense stable clones for uPA significantly reduced invasiveness and tumor formation^19^. A decrease in the migration and invasion of glioblastoma cells expressing antisense MMP9 with the failure to form tumors when injected intracranially in the nude mice has been demonstrated^19^. Interestingly, after knocking down of the uPA in the breast carcinoma MDA-MB-231 by siRNA, *in vitro* migration of the cells was effectively decreased along with a significant reduction in the extracellular MMP9 activities^9^.

Cancer is the multistep complex process and thus blocking of one target is unable to achieve desirable effect. On the basis of these previous studies, indicating the major roles of the uPA and MMP9 in cancer cell progression and tumor growth, we are hypothesizing the synergistic blocking of uPA and MMP9 can reduce tumor aggressiveness and invasion. This may be achieved by increasing the cell–cell adhesion by the expression of major adhesive protein for epithelial characters E –cadherin and down regulating the expression of mesenchymal markers. To test our hypothesis we have knockdown uPA and MMP9 by siRNA in breast cancer cells and examined the cellular behavior and the expression of these key EMT markers. Finally we have reviewed our model in the breast tumor samples expressing the different level of uPA and MMP9.

## Results

### Silencing of uPA and MMP9

After transfecting the breast cancer cells MDA-MB-231, T47D and ZR-75-1 with siRNA against uPA and MMP9, transcript levels have been checked in the respective cells. A significant reduction of the expression of uPA and MMP9 were observed in the MDA-MB-231 cells (80%) in comparison to the T47D and ZR-75-1 cells (Supplementary Fig. 1, Supplementary Table S1). As the MDA MB 231 cells show maximum knock down of both the uPA and MMP9 genes, in the subsequent experiment MDA MB 231 cell line has been used. MTT assay was carried out to examine any non specific cytotoxic effect of the lipid transfection reagent. There was no significant toxic effect observed (Supplementary Fig. S2).

### Effect on the cell cycle

Flowcytometric analysis of the cells transfected with siRNAs was carried out using Propidium Iodide. After 72 hours of transfection, a significant number of the MDA MB 231 cells transfected with both uPA and MMP9, were arrested more in the S and G2-M phase (Supplementary Fig. 3). In the control cells, approximately 16% and 63% of the cells were in the G0 and G0/G1 phase, 8% in the G2/M phase and 12% in the S phase. The number of cells in the G2/M phase was increased to 11% in both the uPA single and MMP9 single transfected cells respectively and 13% in the combined siRNA transfected cells. The number of cells in the S phase was also increased to 16%, 17% and 18% in the uPA single, MMP9 single and combined siRNA transfected cells respectively.

### Effect on the healing capacity of the cells

**Figure 1:**
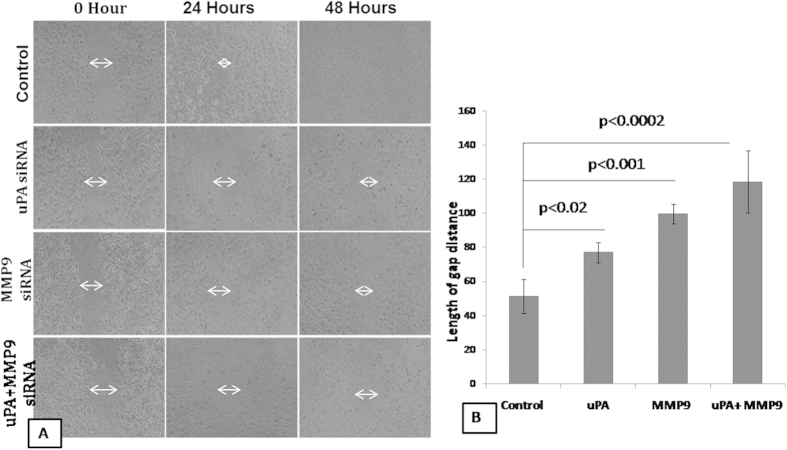
Wound healing capacity of the MDA-MB-231 cells after transfecting with uPA and MMP9 siRNA alone or in combination. (**A**) The wounded area was photographed under phase contrast microscope at 0, 24 and 48 hours after scratching. The length of gap distance was measured at 0, 24 and 48 hours using Image J software (**B**) Bar graph representing the 48 hours post recovery gap distance after induced scratch in the cell culture dish. Control indicates the cells were not transfected with either of the uPA and MMP9 siRNA. (Data are representative of three independently performed experiments and are expressed as mean ± SD).

As uPA and MMP9 has been known to degrade the ECM and aids the cells in migration, we first checked the expression level of the uPA and MMP9 in different breast cancer cell lines. Our, result indicates that the expression levels of the uPA and MMP9 was highest in the MDA-MB-231 cells. The cell line with the highest expression of uPA and MMP9 was tested for the metastatic potential using the scratch wound healing assay after successful down-regulation of the uPA and MMP9 genes. The wounded area in the control cells started to heal up after 24 hours of scratching while the wound in the transfected cells heal up at a very slow rate with almost negligible decrease in the denuded area indicating a low metastatic capacity of the cells in the transfected cells (Fig. 1A). The wound in the control cells almost healed after 48 hours of scratching while wound of the transfected cells failed to heal up even after 48 h. Further analysis using the ImageJ software revealed that the average distance covered by the combined transfected cells were much lesser than that of the cells transfected with uPA and MMP9 siRNA alone and control (Fig. 1B).

### Effect on the migratory and invasive capacity of the breast cancer cells

**Figure 2:**
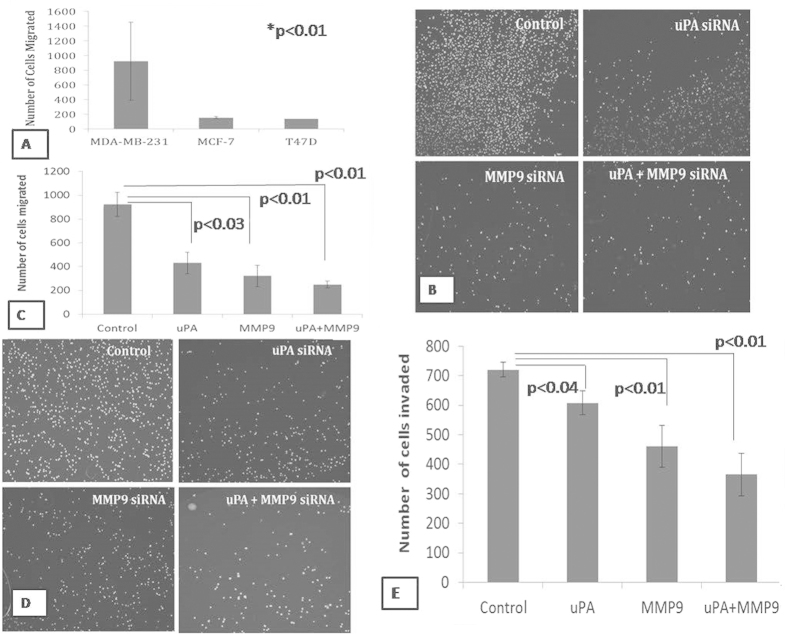
Cells were allowed to migrate in the transwell chamber under chemotactic condition and the migrated cells were stained with DAPI and counted under fluorescence microscope. (**A**) Bargraph showing comparative migratory ability of the MDA-MB-231, MCF-7, and T47D cells (**B**) MDA-MB-231 cells were transfected with uPA and MMP9 siRNA either singly or in combination and allowed to migrate through the membrane of the transwell unit for 18 hrs under chemotactic condition. Migrated cells were stained with DAPI. The photomicrographs were taken under fluorescence microscope using UV filter (**C**) The migrated cells were counted under fluorescence microscope (**D**) Cell after transfection with respective siRNAs were allowed to invade through the matrigel coated membrane of the transwell unit for 22 hrs and stained with DAPI. Photomicrographs were taken under fluorescence microscope using UV filter (**E**) The number of the invaded cells was then counted. Control indicates the cells were not transfected with either of the uPA and MMP9 siRNA. (Data are representative of three independently performed experiments and are expressed as mean ± SD).

With relation to the expression of uPA and MMP9, migratory capacities of the untransfected cells were examined. It was observed that MDA MB 231 cells migrate significantly higher than the other adenocarcinoma cell MCF 7 and ductal carcinoma cell T47D (Fig. 2A). To test whether the down-regulation of the uPA and MMP9 genes change the migratory capacity of the MDA-MB-231 cells, uPA and MMP9 genes were knock down and allowed to migrate and invade further. As shown in Fig. 2B,C, MDA-MB-231 cells demonstrated a significant decrease in the rate of migration. However, the cells that were transfected with both the uPA and MMP9 siRNA, migrate at a remarkably decreased rate when compared with the cells that were transfected singly (Fig. 2B,C). This indicates that the migratory capacity of the mesenchymal cells, MDA-MB-231 has been decreased after the down-regulation of the uPA and MMP9 genes. After successful knockdown of the uPA and MMP9, we have investigated the sustaining invasive capacity of the breast cancer cells. A remarkable decrease in the ability of cellular invasion was observed in comparison to the control cells as evident from the matrigel coated Transwell invasion assay (Fig. 2D). The decrease in the invasive capacity of the cells was more in the combined transfected cells in comparison to the single transfected cells (Fig. 2E).

### Effect on the adhesive capacity of the cells

**Figure 3:**
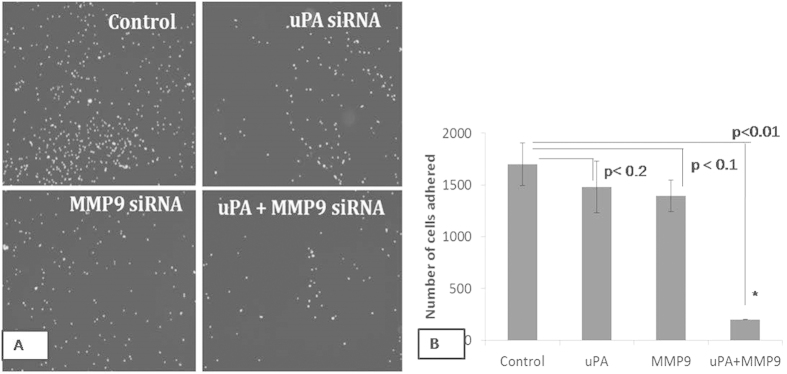
The MDA-MB-231 cells were transfected with respective siRNAs and allowed to adhere on the Matrigel coated wells for 2 hrs. (**A**) Adhered cells were stained with DAPI and photomicrographs were taken under fluorescence microscope using UV filter (**B**) The number of the adhered cells were counted. Control indicates the cells were not transfected with either of the uPA and MMP9 siRNA. (Data are representative of three independently performed experiments and are expressed as mean ± SD).

The adhesive capacity of the metastatic breast cancer cells was tested by seeding the siRNA transfected cells on the Matrigel coated wells. The non-adherent cells were washed off properly with 1XPBS. As seen in Fig. 3A, siRNA mediated down-regulation of the uPA and MMP9 genes decreased the adhesive nature of the cells. The decrease in the adhesive nature was more in the combined transfected cells in comparison to the single transfected cells (Fig. 3B).

### Effect on the reversal of epithelial to mesenchymal transition of the cell

**Figure 4:**
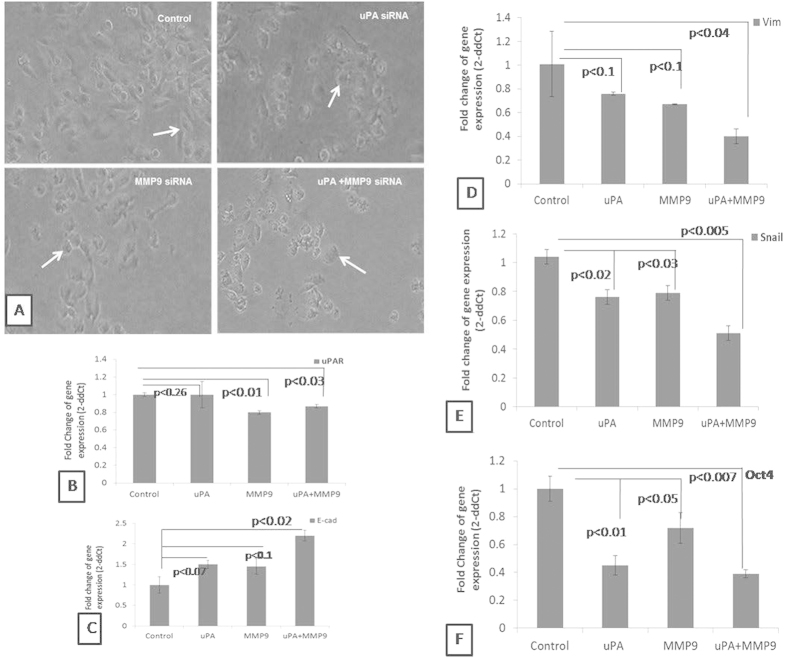
Effect on the reversal of the EMT. MDA-MB-231 Cells were transfected with respective siRNAs and observed after 12 hours for changes in the cellular morphology under phase contrast microscope. (**A**) Control cells are showing elongated spindle shaped appearance. The elongated morphology of the cells started changing towards cobble-stone shape after transfection with respective siRNAs. The cells completely acquired epithelial structure of cobble-stone shape as indicated by the arrow. (Control indicates cells were not transfected with either of the uPA or MMP9 siRNA). After trasfection of siRNA against uPA and MMP9, transcript level (**B**) uPAR (**C**) E-cadherin (**D**) Vimentin (**E**) Snail (**F**) Oct-4 were evaluated by Real time PCR. (uPA means cells were transfected with siRNA against uPA, MMP9 means cells were transfected with siRNA against MMP9 and uPA + MMP9 means cells were transfected simultaneously with siRNA against both MMP9 and uPA). Control indicates the cells were not transfected with either of the uPA and MMP9 siRNA. (Data are representative of three independently performed experiments and are expressed as mean ± SD).

Effect of synergistic knockdown of uPA and MMP9 genes on the reversal of epithelial to mesenchymal transition has been studied. The fibroblast like morphology of the MDA MB **231 **cells were converted into epithelial like structure after knock down of the uPA and MMP9 genes. The control cells appear with an elongated morphology while the siRNA transfected cells appeared as rounded morphology with no extensions. Cells become more rounded losing the characteristic spindle shaped feature of the mesenchymal character and acquiring the rounded cobblestone structure of the epithelial cell type feature. The elongated morphology of the cells were converted towards cobble-stone shape after knock down of uPA or MMP9. The cells were completely acquired epithelial structure of cobble-stone shape after synergistic knockdown of uPA and MMP9 (Fig. 4A).Accordingly, transcript level of different EMT markers like E-cadherin, Vimentin and Snail have been investigated upon knockdown of the uPA and MMP 9. Synergistic knockdown of uPA and MMP9, causes 2.0 fold up-regulation of the epithelial marker E-cadherin (Fig. 4C), and a sharp down-regulation of mesenchymal marker - Vimentin and Snail (Fig. 4D,E).

### Knockdown of MMP9 down regulate the uPAR

It has been examined the effect of silencing of uPA and MMP9 on the expression of uPAR. As shown in the Fig. 4B, there is no effect on the expression of uPAR after silencing of uPA. But knockdown of MMP9, down regulate the expression of uPAR.

### Effect on the expression of the stem cell marker Oct-4

After transfection of siRNA against uPA and MMP9 in combination, there was a significant reduction in the transcript level of Oct-4. After transfection of siRNA against MMP9, there was a minimal reduction while silencing of uPA along with MMP9, causes significant down regulation of Oct-4 (Fig. 4F).

### Expression of uPA and MMP2 and 9 in infiltrating ductal carcinoma of breast tumor

**Figure 5:**
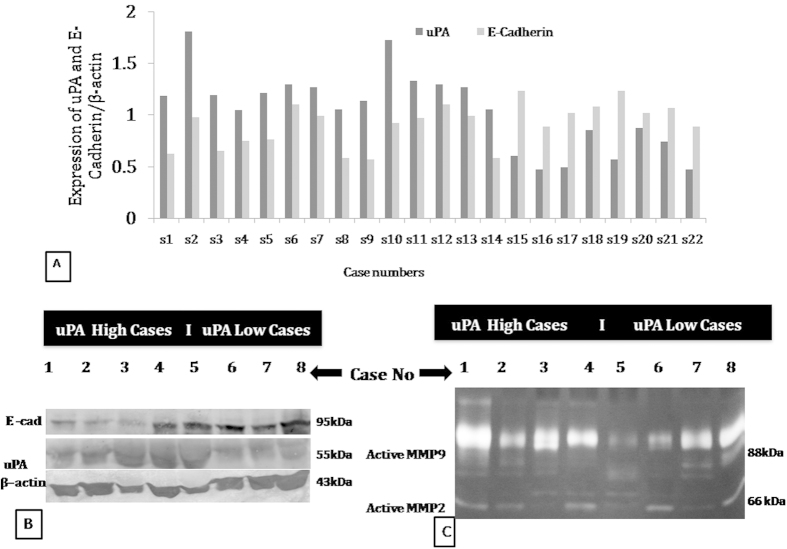
Expression of uPA, E –cadherin, MMP2 and MMP9 in invasive ductal breast tumor. (**A**) Fold change of protein expression of uPA and E-cadherin in terms of the β-actin expression following western blot of the individual 22 tumor samples. (**B**) Representative western blot image showing the sample group based on the uPA high and uPA low expression (**C**) Representative Zymography gel for the expression of MMP9 and MMP2 (1–4, tumors with high uPA expression; 5–8, patients tumors with low uPA expression).

Expression of uPA in the ductal carcinoma tissues was evaluated by the western blot analysis after surgical removal of the tumor (Fig. 5A,B). On the other hand, expression of MMP2 and 9 were evaluated by the gelatin zymography (Fig. 5C). The natures of the expression of these molecules in different clinical conditions are presented in Table 1. The tumors have been classified in accordance to the higher and lower expression of uPA, irrespective of the stage and grade of the tumors. It has been observed that the tumor expressing higher level of uPA also expresses higher level of both MMP 2 and 9. Surprisingly, the tumors expressing low level of uPA also exhibit very low level of the active MMP9 (Fig. 5C).

### Expression of E-cadherin in correlation with uPA in human breast tumor tissues

Western blotting was carried out to study the expression level of E-cadherin in a cohort of 22 patients selected for the study. Interestingly, in most of the cases it has been observed that the patients expressing high uPA and MMP 9 expresses less of the E-cadherin, and (Fig. 5). On the other hand, the tumor expressing low uPA and MMP 9 expresses more of E-cadherin Table 1.

### Expression of the Mesenchymal marker Vimentin in human breast tumor tissues

**Figure 6:**
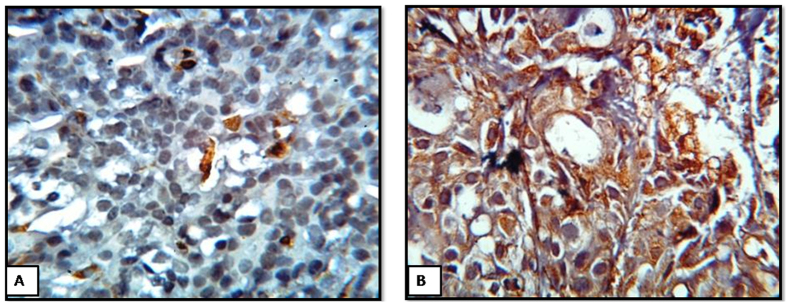
Expression of the mesenchymal marker Vimentin in the duct lobular area of the invasive breast cancer tissue. (**A**,**B**). IHC performed on 5 μm paraffin-embedded sections of breast tumor using a polyclonal antibody to Vimentin. (**A**) The vimentin antibody showed no reactivity with the ductal component (**B**) The vimentin antibody showed cytoplasmic reactivity with the ductal component.

Further, the expression of vimentin was studied in the breast cancer tissues by immunohistochemistry. It has been observed that in some of the patients vimentin, has been expressed in the ductal component of the invasive breast cancer tissues (Fig. 6).

## Discussion

In our present work, two key molecules for metastasis, uPA and MMP9 have been knockdown in the breast cancer cells to check the cancer progression. Thus, using siRNA against the uPA and MMP9, nearly 80–90% transcripts have been silenced, without exerting any toxic effect to the cells.

From our study, it has been revealed that down-regulation of the uPA and MMP9 arrested the cells for further growth. A decrease in the migratory and invasive capacities of the transfected cells was observed. The decrease was more in the combined transfected cells than in the control cells. Wound healing assay was also carried out after transfecting the cells with the siRNA against uPA and MMP9. This wound healing assay or directional cell migration assay mimics the migration of the cells during wound healing *in vivo*^20^. In our present work, it has been observed that wound healing capacity of the transfected cells slowed down in comparison to that of the control cells with least healing up in the combined transfected cells. It should be noted that slow wound healing of the combined transfected cells may be due to the slow directional migration.

It has been reported that binding of uPA to its receptor uPAR transmits signal over the cell membrane that leads to increased cell motility in some cell types^21^. The chemotactic effect of uPA requires binding to the uPAR^2^. In the present work, knockdown of uPA did not change any significant expression of uPAR (Fig. 4B). Interestingly, it has been previously reported that siRNA mediated down-regulation of the uPAR leads to the decrease in the expression level of the uPA but when uPA was down-regulated there was no change in the expression of the uPAR in the breast cancer cells, as the expression of the uPA is regulated transcriptionally by uPAR^9^. It has also been reported that uPA is activated after it binds to uPAR and that the inhibition of uPAR reduces the active uPA^7^. On the contrary, in our present work when MMP9 was knockdown, there was a decrease in the expression of uPAR (Fig. 5A). This is in correlation with the previous findings that when MMP9 was inhibited using siRNA, there was a reduction in the expression level of uPAR of about 60% in MDA MB 231 and 40% in ZR 75-1 cells^7^. In the simultaneously transfected cells the expression level of uPAR was decreased in comparison to that of the control cells. The binding of the uPA to uPAR leads to the activation of variety of signaling molecules. Many of such signaling cascades control the cell migration and invasion along with the survival and cellular migration and metastasis^9^,^19^,^22^. It has been reported that MMP9 is also involved in cancer cell migration^7^. Our results thus correlate with the previous findings, that blocking of the uPA and/or uPAR functions, inhibited breast cancer invasion and metastasis^22^.

In the present study, uPA and MMP9 transfected cells were seeded on the matrigel coated wells and allowed to adhere for nearly 2 hours. The transfected cells adhered but less in comparison to that of the control cells. Interestingly, the number of adhered cells was least in the combined transfected cells compared to the single transfected cells. It has been reported that MMP9 activates some of the signaling pathways involved in cellular adhesion^8^,^9^. Down-regulation of the expression of the uPA decreases interaction of the uPAR and integrins of the vitronectin receptor of the ECM^19^. Other than this, decrease in the MMP9 might have failed to activate the signaling pathways responsible for the cellular adhesion. The decrease in the adhesive property of the cells to the ECM prevents the cells from interacting with the ECM and ultimately the cells lose their adhesive property significantly in the synergistically transfected cells.

**Figure 7:**
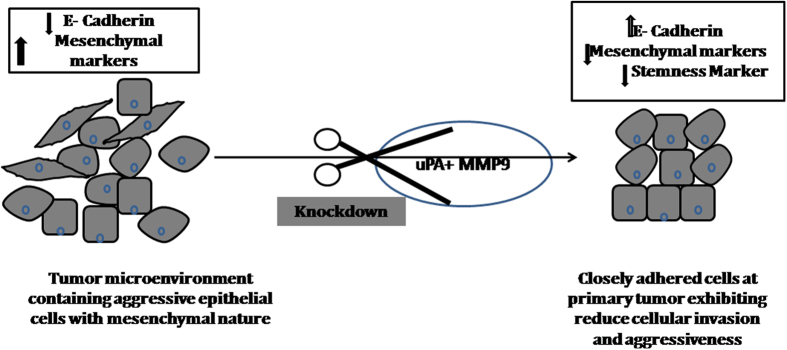
Schematic diagram showing the model of simultaneous knockdown of uPA and MMP9 in breast tumor. The tumor micro-environment is represented by the presence heterogeneous cell populations – mainly epithelial cells with mesenchymal nature along with other cell types. Aggressive tumor with invasive cells may be dislodged by the reduce expression of E-cadherin. Thus, after simultaneous knockdown of uPA and MMP9, higher expression of E-cadherin and lowering of mesenchymal and stem cell marker may reduce the cellular invasion and tumor aggressiveness.

Major hallmark for the tumor aggressiveness is the loss of E-cadherin. Loss of E-cadherin help cells to dislodge from the primary tumor site and make cell invasive with the help of other molecules, specially release of the proteases to degrade the basement membrane. Thus, it has been hypothesized in the present work, that combined knockdown of uPA and MMP9 can collectively increase the expression of cell to cell adhesion molecule: E-cadherin and changes the expression of the other genes responsible for the “epithelial to mesenchymal transitions”. Accordingly, we have studied the expression of the key genes of the, EMT: epithelial marker E-cadherin^4^ and mesenchymal markers Vimentin^4^ and Snail^23^. Thus, it has been observed that there is an increase in the expression of E-cadherin and a decrease in the expression of Vimentin and Snail. It has already been reported that, Snail is one of the transcriptional repressors of E-cadherin expression^4^. It has been reported that breast tumor consisting of a heterogenous cell population in which some of the cells acquire the property of stemness that contributes to negative prognosis^5^. Thus, to see the changes of the stemness of the breast cancer cells, we have studied the expression of Oct-4. Oct-4 is a transcription factor required for maintaining self-renewal in the stem cells^6^. In the present study, it has been observed very promisingly, that synergistic knock down of the uPA and MMP 9 decrease the expression of Oct-4. Wang *et al*.^24^ reported that, after simultaneous down-regulation of the Oct-4 and Nanog there was an up-regulation of E-cadherin and a decrease in the expression of Vimentin, Slug and Snail. While in our study, it has been observed that, with the decrease in the expression level of Oct-4 after the down-regulation of uPA and MMP9 there was a decrease in the expression level of Vimentin and Snail with an increase in the E-cadherin expression. Thus, together with the functional characteristics, molecular evidence suggest that combined silencing of uPA and MMP9 can reduce the tumor invasion and aggressiveness by increasing the expression of E-cadherin and reducing the mesenchymal markers (Fig. 7).

To review the translational potential of our *in vitro* model, human invasive breast tumor tissues were collected and checked the expression of uPA, MMP9, E-cadherin along with, mesenchymal marker Vimentin. Very promisingly it has been observed that the expression of uPA and MMP9 are well correlated with expression of E-cadherin in individual patients. In most of the cases, tumors expressing higher level of uPA and MMP9 show lower expression of E cadherin. To address the expression of mesenchymal marker, we have also studied the expression of vimentin. Generally, vimentin is expressed in the stromal part of the breast^25^ as the stromal part of the breast is of mesenchymal origin. But we have chosen for observing the expression of vimentin in the ductal part of the tumor, which is of epithelial origin. This is to review the tumor aggressiveness associated with epithelial to mesenchymal transition. In the present investigation, very few cases show ductal cytoplasmic positive for vimentin. But in most of the cases, negative for vimentin expression was found in the ductal component of the tumor. This apparent discrepancy in the expression of vimentin and EMT may not be unusual. The observation in relation with the negative expression of vimentin in the ductal area of the invasive tumor shows that tumor cells in question are not fully converted to mesechymal type, rather still maintaining epithelial type, but they are aggressive and invasive. Thus invasive breast tumor consisting of heterogeneous cell population, mostly represented by epithelial cells, epithelial cells with mesenchymal nature, CSCs, with few percentage of epithelial cells converting to mesenchymal cells. Our observation is clearly supported by the findings of the hetrogenous nature of circulating tumor cells by Mego *et al*., Alix-Panabières and Pantel, and Satelli *et al*.^26^,^27^,^28^, which are the root of the metastasis. Thus, the findings of the present work advocate the concept of epithelial cells with mesenchymal nature for tumor aggressiveness and invasion rather than ‘epithelial to mesenchymal transition’.

Based on the *in vitro* cell culture study along with the evidence of the expression correlation in breast tumor tissue, the present investigation clearly shows the simultaneous inhibition of uPA and MMP9 can inhibit tumor invasion and aggressiveness. This model can be utilized for the adjuvant therapy or combination therapy for the treatment of invasive breast cancer or to control the primary breast cancer.

## Methods

### Cell Culture

Human breast cancer cells MDA-MB-231, MCF-7, T47D and ZR-75-1 were obtained from National cell repository facility of India - National Center for Cell Science, Pune, India, which were originally procured from American Type Culture Collection, USA. Cells once procured, were kept in cultures and used within 1–2 months for the different experiments. MDA-MB-231 cells were grown in L-15 medium (Himedia, India) supplemented with L-Glutamine. MCF-7 cells were grown in DMEM medium (Himedia, India). T47D and ZR-75-1 cells were grown in RPMI-1640. All the media in which the cells were grown contained 10% FBS (GIBCO) and 1% L-Glutamine-Penicillin-Streptomycin (200 mM L-Glutamine, 10,000 units/mL Penicillin and 10 mg/mL Streptomycin) (Himedia, India). The MCF-7, T47D and ZR-75-1 cells were maintained at 37 °C in a humidified incubator with CO_2_ while MDA-MB-231 cells were maintained at 37 °C in a humidified incubator without CO_2_.

### siRNA transfection

Predesigned siRNA against uPA and MMP9 were procured from Ambion by life technologies, The sequences of siRNA against uPA was: 5′-GCUUAACUCCAACACGCAAtt-3′ (S) and 5′-UUGCGUGUUGGAGUUAAGCct-3′ (AS) and while siRNA against MMP9 was 5′-GACCU GGGCAGAUUCCAAAtt-3′(S) and 5′-UUUGGAAUCUGCCCAGGUCtg-3′ (AS). Transfections of the siRNAs were carried out either uPA or MMP9 alone or simultaneously with uPA and MMP9. Cells transfected with GAPDH siRNA was used as a positive control. While scrambled sequence of nucleotides was used as a negative control. 40 × 10^4 ^cells were plated in 35 mm culture dishes for the transfection experiments. When the cells have reached nearly 30–40% confluence, transfection was carried out in an antibiotic free media. Transfections were performed using Interferin trasfection reagent (Poly Plus, France) as per the manufacturer’s instructions. After 24 hours of transfection, media was replaced with fresh antibiotic free media and left inside the incubator at 37 °C for 48 hours. Cells were used for subsequent different experiments. For the assessment of any cytotoxic effect of the Interferin trasfection reagent, MTT assay was carried out. Knockdown efficiencies of the siRNAs were evaluated by estimating the transcript levels of the respective genes by Real Time PCR analysis. Initially, MDA MB 231, MCF 7 and T47D cells were considered for knockdown of the uPA and MMP9 genes. Accordingly, knockdown efficacy had been evaluated. The cell showing highest knockdown efficacy of both the genes had been considered for the different subsequent experiments.

### Cell Cycle analysis

The cells were plated in 35 mm dish with seeding density of 40 × 10^4^ and transfected. After 72 hours of transfection, cells in the supernatant as well as the adherent cells were collected, washed and fixed with cold 80% ethanol. The cells were then incubated with RNase A (Sigma Aldrich) and stained with propidium iodide (Sigma Aldrich) for 30 minutes in the dark. Cells were analyzed with BD FACS Verse (BD Bioscience).

### Wound Healing Assay

The cells were allowed to transfect for 72 hours till the cells were grown to near confluence and then kept serum starved for overnight. On the next day a scratch was made on the cell monolayer using a sterile 200 μl pipette tip to assess the directional motility in healing the “wound”. The degree of wound closure was assessed in three randomly chosen fields. Photographs of the wounds were taken at 0, 24 and 48 hours.

### Transwell migration and invasion Assay

The comparative *in vitro* migratory capacity of the different types of epithelial cells : adinocarcinoma cells - MDA MB 231 and MCF 7 and ductal carcinoma cells T47D, were checked in accordance with the expression of uPA and MMP9. The MDA MB 231 cells were transfected with siRNA for uPA and MMP9. After 72 hours of transfection the cells were trypsinised and added in the serum free media in the upper chamber of the cell migration insert (polyethylene teraphthalate membranes; pore size 8 μm; Becton Dickinson) with a seeding density of 5 × 10^4 ^cells. FBS containing media was placed in the lower chamber and allowed to migrate for 18 hours. For the invasion assay, the transwell units were coated with 100 μl Matrigel. The transfected cells were allowed to invade through the Matrigel for 22 hours under the chemotactic drive. After the incubation period, the non-migrated or the non-invaded cells in the upper compartment were wiped off gently using a cotton swab. The migrated or the invaded cells on the lower surface were fixed with 100% methanol and stained with DAPI. The number of migrated or invaded cells in four quadrants were observed under fluorescence microscope and counted. Quantitation of the migrated and invaded cells was done in triplicates and each experiment was repeated thrice.

### Cell adhesion assay

The *in vitro* cellular adhesion assay of the uPA and MMP9 siRNA transfected cells was studied by seeding the cells on the Matrigel (Sigma Aldrich) coated wells. Transfected cells were seeded with a seeding density of 5 × 10^4^ cells in the serum free media. The cells were allowed to adhere for 2 hours on the matrigel. After washing twice with 1XPBS, the adhered cells were fixed with 100% methanol and stained with DAPI. The number of adhered cells were then observed under fluorescence microscope and counted in four independent fields.

### Real Time PCR

Real time PCR was carried out to study the change in expression of the different genes after knock down of uPA and MMP9. Total RNA was extracted from the control and transfected cells using TRIzol reagent (Invitrogen). Isolated RNA was reverse transcribed using Verso cDNA synthesis kit (Thermo Scientific) according to the manufacturer’s protocol. PCR amplification was carried out using the SYBR green method with the help of Light Cycler 480 machine (Roche). GAPDH was used as a reference gene for normalization. The PCR conditions consisted of 1 cycle of initial activation at 95 °C for 10 minutes, then 40 cycles of denaturation at 95 °C for 10 seconds, annealing at 50 °C for 20 seconds and extension at 72 °C for 15 seconds, followed by melting reaction step of 65 °C and 95 °C for 1 cycle. Relative changes in the gene expression were analyzed using the 2^-ΔΔCT^ method. The sequences of the primers used are presented at Supplementary Table S2.

### Breast tumor tissue samples

To review the expression pattern of E-cadherin in relation with uPA and MMP9, and also the concept of ‘epithelial to mesenchymal transition’ in breast tumor, surgically resected tumor samples have been included. The study was carried out as per Helsinki Declaration and duly approved by the Institutional Ethical Committee of Burdwan Medical College and Hospital and The University of Burdwan. Patients pathologically diagonosed as invasive ductal carcinoma, without the history of any type of neoadjuvant therapy or radiotherapy were included in the study. Clinicopathological parameters were thoroughly studied by two experienced pathologists. Tumor samples have been broadly categorized in two groups: tumor with both higher expression of uPA and active MMP9 and tumor with lower both expression of uPA and active MMP9. To study the tumor aggressiveness, expression of E-cadherin in these two tumor groups have been examined by western blotting. The expression of the mesenchymal marker - vimentin in the ductal area of the breast tumors, has been studied by immunohistochemistry.

### Western Blotting

For the expression of uPA and E-cadherin in the tumor tissue as stated above, western blotting was carried out. The tissues were homogenized properly with RIPA buffer and 30 μg of tissue homogenate per sample were loaded on SDS-PAGE gels. The gels were blotted using the polyvinylidene fluoride (PVDF) membrane (Sigma) and then subsequently blocked with the blocking buffer consisting of concentrated buffered saline containing detergent and casein as provided in the kit, according to the manufacturer’s instruction. The blocked membrane was incubated with E-cadherin, uPA and β-actin primary antibodies as per respective dilution. Immunoreactive proteins were detected with Alkaline Phosphatase (ALP) conjugated secondary antibody provided in the kit. NBT/BCIP chromogenic substrate has been used to detect the immunoreactive bands and then quantified by densitometry.

### Gelatin Zymography

The expressions of MMP 9 along with MMP2 in the tumor samples were studied by Gelatin Zymography. The tissue homogenate was loaded in each well of the 10% SDS gel containing 15 mg/ml gelatin. After electrophoresis, SDS was removed from the gel by washing with buffer containing 2.7% (v/v) Triton-X 100 (pH 8.0) for half an hour at room temperature followed by washing in distilled water for three times. Gelatinase activity was developed for 48 hrs at room temperature in a buffer containing 0.5 M Tris-HCl, 2.0 M NaCl, 0.05 M CaCl_2_, 0.2% Brij 35 (v/v) (pH7.8). Later the gel was stained with 0.1% Coomassie Brilliant Blue R-250 (w/v) in 50% methanol, 10% acetic acid and destained with 20% methanol, 10% acetic acid. Gelatinolytic activity was observed as clear bands against the blue background. Human recombinant MMP-2 (10 ng) was loaded as a reference standard for calculating densitometric value.

### Immunohistochemistry

Surgically resected primary breast tumor tissues after collection were fixed in 10% formalin, embedded in paraffin and sectioned (5 μm). The sections were used for the localization of Vimentin. Immunostaining was performed using the Leica Microsystems IHC kit following the manufacturer’s protocol. Briefly, after the de-paraffinization and rehydration, heat induced antigen retrieval was performed for 10 min prior to peroxidase blocking. The sections were then incubated with primary (rabbit) polyclonal antibody against Vimentin for 60 mins. Second antibody conjugated HRP system was added to the sections, and incubated for another 60 minutes. DAB substrate reagent was added as per manufacturer protocol. The sections were counterstained with Harris’s haematoxylin, dried and mounted with coverslip. The expression of vimentin in the duct-lobular area has been studied under light microscope.

### Image analysis

Cell cycle analysis was performed by BD FACSVerse flow cytometer with BD FACSuite software. Wound healing image was captured by Olympus Inverted phase contrast microscope attached with Magnus MIPS USB2.0 camera, subsequent gap distance was analysed by Image J software. For migration, invasion and adhesion assay DAPI stained cells were imaged by Leica DMI 6000B Fluorescence Microscope attached with Leica DFC 450C camera.

### Statistical Analysis

Results of the experiments were statistically evaluated using ANOVA followed by Student’s t-test. A p-value < 0.05 was considered for statistically significant. All experiments of the *in vitro* cell culture were carried out in triplicate and repeated at least three times. The χ^2^ Contingency tables were used to analyze the association of the expression of E-cadherin, vimentin with uPA, MMP9 and different clinical-pathological parameters related with breast tumor samples. The results were analyzed using GraphPad Prism software.

## Additional Information

**How to cite this article**: Moirangthem, A. *et al*. Simultaneous knockdown of uPA and MMP9 can reduce breast cancer progression by increasing cell-cell adhesion and modulating EMT genes. *Sci. Rep.* **6**, 21903; doi: 10.1038/srep21903 (2016).

## Supplementary Material

Supplementary Information

## Figures and Tables

**Table 1:** Expression of epithelial marker E-cadherin and mesenchymal marker Vimentin along with different clinicopathological parameters in the ductal carcinoma of breast in relation with uPA and MMP9.

Characteristics	Vimentin (in ductal compartment)			E-cadherin			
	Negative	Positive	p value	Low	High	Total	p value
Age							
≤50 years	17	2	0.37	14	6	20	0.53
>50 years	2	1		1	2	3	
Tumor Size							
≤2 cm	0	0	NA	0	0	0	NA
>2 cm	19	3		16	6	22	
Lymph node status							
Negative	9	0	0.24	6	3	9	0.647
Positive	10	3		8	5	13	
Histological Grade							
1	6	0	0.52	4	2	6	0.615
2	9	2		8	5	13	
3	4	1		2	1	3	
uPA							
Low	8	0	0.27	2	6	8	0.001
High	11	3		12	2	14	
Active MMP9							
Low	8	0	0.27	1	7	8	0.008
High	11	3		13	1	14	
