# Supplementary material for: Simultaneous knockdown of uPA and MMP9 can reduce breast cancer progression by increasing cell-cell adhesion and modulating EMT genes
Source: Sci Rep. 2016 Feb 24;6:21903. doi: 10.1038/srep21903 (PMC4764826; doi:10.1038/srep21903)
Supplement: Supplementary Information [file srep21903-s1.doc]

**Supplementary File: _SREP_21903**

**Simultaneous knockdown of uPA and MMP9 can reduce breast cancer progression by increasing cell-cell adhesion and modulating EMT genes**

*Anuradha Moirangthem^1^, Banashree Bondhopadhyay^1^, Mala Mukherjee^2^, Arghya Bandyopadhyay^2^ , Narendranath Mukherjee^3^, Karabi Konar^2^, Shubham Bhattacharya^2^ Anupam Basu^1^*^*^,

^1^Molecular Biology and Human Genetics Laboratory, Department of Zoology, The University of Burdwan, Golapbag, Burdwan 713104, West Bengal, India. ^2^Department of Pathology, ^3^Department of Surgery, Burdwan Medical College and Hospital, Burdwan.

**^*^ Correspondence:** abasu@zoo.buruniv.ac.in

^1^Molecular Biology and Human Genetics Laboratory,

Department of Zoology,

The University of Burdwan,

Golapbag, Burdwan 713104,

West Bengal, India

Ph No. + 919734029333

**Supplementary Tables**

**Table S1** Relative expression of uPA and MMP9 as measured by Real Time PCR. Relative expression was calculated considering the expression of housekeeping gene GAPDH as 100% expression. Values inside the parenthesis denote the mean Ct value.

| **Cell Line** | **% Expression uPA (Ct)** | **% Expression MMP9 (Ct)** | **% Expression GAPDH (Ct)** |
| --- | --- | --- | --- |
| ZR-75-1 | 42.44 (33.31) | 41.79 (33.83) | 100(14.14) |
| T47D | 54.28(28.92) | 45.31 (34.65) | 100(15.7) |
| MDA-MB-231 | 73.98(19.53) | 56.11 (25.75) | 100(14.45) |

**Table S2: Sequences of the primers used for Real Time PCR**

| **Primer** | **Sequence** |
| --- | --- |
| uPA Forward | 5’- CTGTGAAATAGATAAGTCAAAAACC-3’ |
| uPA Reverse | 5’- TGTCTGGGTTCTGCAGTAATT-3’ |
| MMP9 Forward | 5’- CCGGACCAAGGATACAGTTT-3’ |
| MMP9 Reverse | 5’- CTCAGAGAATCGCCAGTACTTC-3’ |
| uPAR Forward | 5’- GGATCCAGGAAGGTGAAGAA-3’ |
| uPAR Reverse | 5’- TTGTTGTGGAAACCATTGGA-3’ |
| E-cadherin Forward | 5’- TCCCACCACGTACAAGGGTC-3’ |
| E-cadherin Reverse | 5’- GTGTATACAGCCTCCCACGC-3’ |
| Vimentin Forward | 5’- AAGACACTATTGGCCGCCTG-3’ |
| Vimentin Reverse | 5’- CAGAGAAATCCTGCTCTCCTCG-3’ |
| Snail Forward | 5’- TAGCGAGTGGTTCTTCTGCG-3’ |
| Snail Reverse | 5’- TGCTGGAAGGTAAACTCTGGAT-3’ |
| Oct-4 Forward | 5’- CTTCAGGAGATATGCAAAGCAGAA-3’ |
| Oct-4 Reverse | 5’- TGCTGGAAGGTAAACTCTGGAT-3’ |
| GAPDH Forward | 5’- TTCGACAGTCAGCCGCATCTTCTT-3’ |
| GAPDH Reverse | 5’- GCCCAATACGACCAAATCCGTTGA-3’ |

**Supplementary Figures**


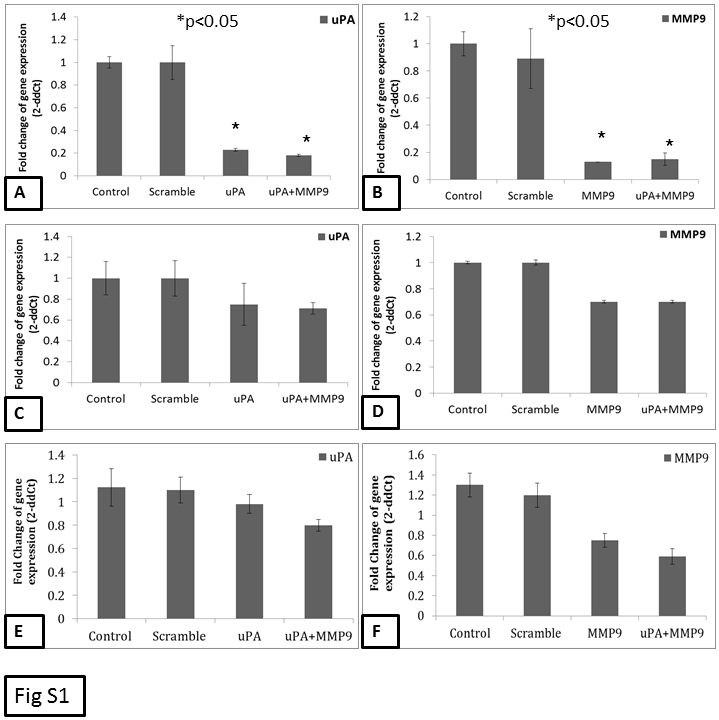


**Supplementary Figure S1.** Cells were transfected with uPA and MMP9 siRNA either alone or combined. Knockdown efficacies of the siRNAs after 48 hours of the transfection were evaluated by the real time PCR for change of the respective transcripts level in (A-B) MDA-MB-231 cells, (C-D) T47D cells, (E-F) ZR 75-1 cells. Fold change of expression was calculated by 2^-ΔΔCt^ method. Control indicates the cells were not transfected with either of the uPA and MMP9 siRNA. Scramble means, cells were transfected with non specific scramble siRNA. (Data are representative of three independently performed experiments and are expressed as mean ± SD).


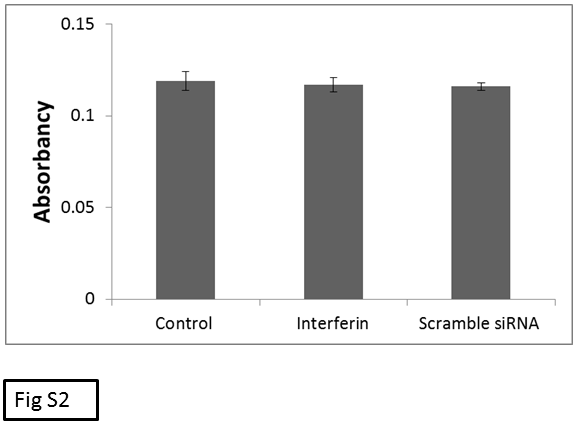


**Supplementary Figure S2.**  MDA MB 231 cells were transfected with Interferin alone and Scramble siRNA for 72 hours to assess the cytotoxic effect using MTT. (Control indicates cells were incubated with complete medium without antibiotic), Interferin, means cells were incubated with lipid (Inteferin) containing transfection medium without any siRNAs, Scrambled means cells were incubated with scrambled siRNA). (Data are representative of three independently performed experiments and are expressed as mean ± SD).


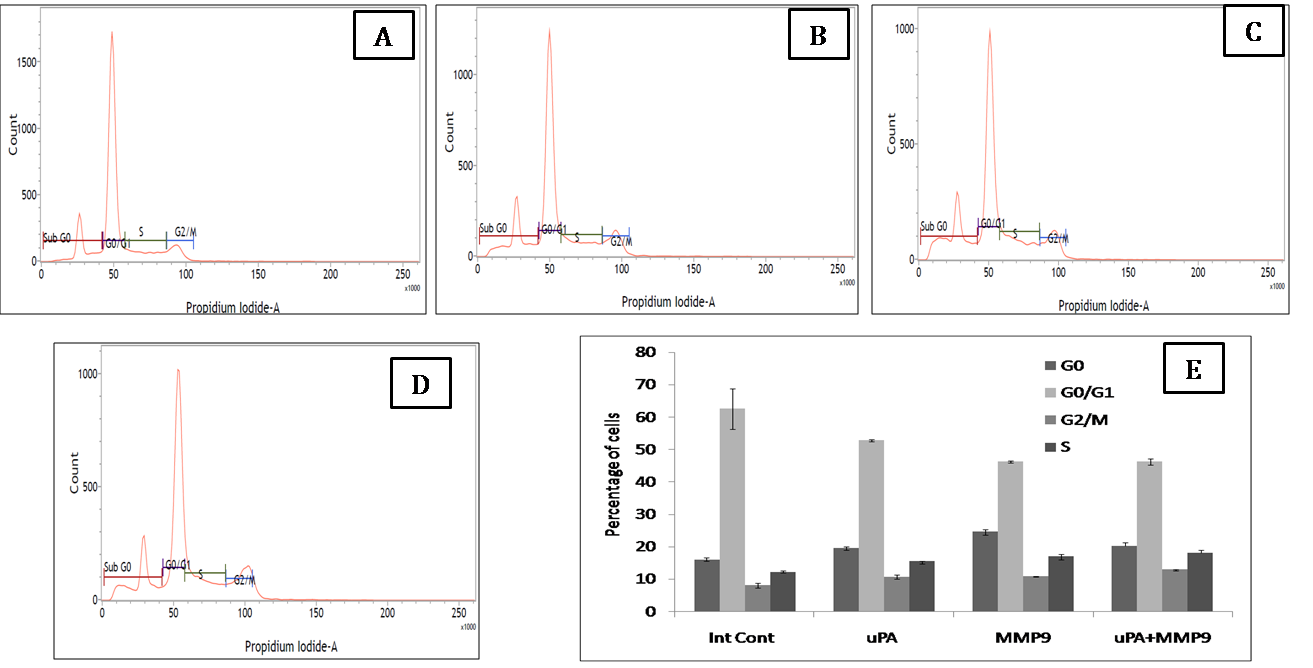


**Supplementary Figure S3.** MDA-MB-231 cells were transfected with respective siRNAs for 72 hours. After transfection, cells were fixed and stained with PI and analyzed in BD FACS Verse Flow cytotmeter for cell cycle analysis. (A) Control cells, (B) cells transfected with siRNA against uPA (C) cells transfected with siRNA against MMP9 (D) cells transfected with siRNA against both uPA and MMP9 (E) Bar graph showing the distribution of the cells in different phases of cell cycle. Control indicates the cells were not transfected with either of the uPA and MMP9 siRNA. (Data are representative of three independently performed experiments and are expressed as mean ± SD).
